# Cytokinin oxidase gene *CKX5* is modulated in the immunity of *Arabidopsis* to *Botrytis cinerea*

**DOI:** 10.1371/journal.pone.0298260

**Published:** 2024-03-13

**Authors:** Ruolin Wang, Beibei Li, Jiang Zhang, Ling Chang

**Affiliations:** State Key Laboratory of Biocatalysis and Enzyme Engineering, School of Life Sciences, Hubei University, Wuhan, China; University of Agriculture Faisalabad, PAKISTAN

## Abstract

In our previous work, cytokinin (CK) signaling and biosynthesis were found to be modulated during *Arabidopsis* defense against infection by the necrotrophic pathogen *Botrytis cinerea*. Notably, the expression level of *CYTOKININ OXIDASE/DEHYDROGENASE 5* (*CKX5*) was significantly induced in *B*. *cinerea*-infected leaves and later in distant *B*. *cinerea*-untreated leaves of the same plant. To confirm and determine how *CKX5* is involved in the response to *B*. *cinerea* infection, transcript levels of *CKX* family genes were analyzed in *B*. *cinerea*-inoculated leaves, and only *CKX5* was remarkably induced by *B*. *cinerea* infection. Furthermore, *CKX5*-overexpressing *Arabidopsis* plants were more resistant to *B*. *cinerea* than wild-type plants. Transcription factors (TFs) binding to the *CKX5* promoter were then screened by yeast one-hybrid assays. Quantitative Real-Time Reverse Transcription PCR (qRT-PCR) analysis further showed that genes encoding TFs, including *WRKY40*, *WRKY33*, *ERF6*, *AHL15*, *AHL17*, *ANAC003*, *TCP13* and *ANAC019*, were also strongly induced in infected leaves, similar to *CKX5*. Analysis of *ERF6*-overexpressing plants and *ERF6*-and *AHL15*-knockout mutants indicated that *ERF6* and *AHL15* are involved in plant immunity to *B*. *cinerea*. Furthermore, *CKX5* upregulation by *B*. *cinerea* infection was affected when *ERF6* or *AHL1*5 levels were altered. Our work suggests that *CKX5* levels are controlled by the plant defense system to defend against attack by the pathogen *B*. *cinerea*.

## Introduction

Plants have evolved a concerted suite of defense systems involving phytohormone pathways in response to pathogen attack, among which is the backbone formed by jasmonate/ethylene (JA/ET) and salicylic acid (SA) [1,2]. Additionally, cytokinins (CKs), essential hormones controlling almost every aspect of plant growth and development, are known to be important in both the defense response and pathogen virulence [3,4]. In some cases, (hemi)biotrophic pathogens can secrete CKs or induce CK production in the host plant to achieve pathogenesis. The hemibiotropic pathogen *Rhodococcus fascians* secretes a combination of various CK species, which are recognized by CK receptors in *Arabidopsis* and cause proliferation of young shoot tissues into differentiated leafy galls [5]. In addition, it has been suggested that CK-secreting biotrophs or hemibiotrophs manipulate CK signaling to regulate the host cell cycle and nutrition allocation [6]. Conversely, plant immunity uses CKs to resist pathogen infection through different mechanisms. It has been suggested that host-derived CKs influence plant immunity in an SA-dependent manner based on the finding that the cytokinin-activated transcription factor ARR2 interacts with TGA3, a transcription factor of SA-responsive genes, to regulate defense-related genes in response to biotrophic infection [7]. On the other hand, CKs mediate resistance against *Pseudomonas syringae* in tobacco by increasing antimicrobial phytoalexin synthesis independent of SA signaling [8].

Studies concerning the role of CKs in plant interactions with necrotrophic pathogens such as *B*. *cinerea*, which does not produce detectable CKs [9,10], are less abundant and more complex. Expression of the CK biosynthesis gene *IPT* under control of the *SAG12* (senescence-specific gene) promoter results in increased resistance of *Arabidopsis thaliana* to *B*. *cinerea* infection [11]. Gupta et al. [12] found that CK promotes the resistance of tomato to *B*. *cinerea* through an SA- and ET-dependent mechanism. We recently reported that CK responds to *B*. *cinerea* infection in a variety of ways that are differentially modulated by the JA and ET pathways in *Arabidopsis* [13]. More recently, CKs have been described as having a dual role in plant-*B*. *cinerea* interactions, inhibiting *B*. *cinerea* development and virulence while positively regulating *B*. *cinerea* energy utilization [14,15]. In our previous work, we found that the expression level of *CYTOKININ OXIDASE/DEHYDROGENASE 5* (*CKX5*), which encodes a CK degrading protein, was markedly induced in locally infected leaves and distant leaves of the same plant without pathogen inoculation [13]. To explore the mechanism by which *CKX5* is strongly upregulated after *B*. *cinerea* infection, we performed yeast one-hybrid (Y1H) assays to reveal the transcription factors (TFs) that bind to the *CKX5* promoter, and we preliminarily investigated relationships between the screened TFs and *CKX5* during *B*. *cinerea* infection in *Arabidopsis*.

## Materials and methods

### Plant material and growth conditions

All *Arabidopsis thaliana* plants used in this study were of the Columbia-0 (Col-0) ecotype. Seeds of *erf6* (SALK_087356, NASC stock number: N2103286), *ERF6* overexpressing line (TPT_4.17490.1A, NASC stock number: N2102191) and *ahl15* (SALK_040729C, NASC stock number: N668128) were obtained from the Arabidopsis seeds share center (Arashare, https://www.arashare.cn/). Detailed information about these seeds can be found on the website of Arabidopsis Biological Resource Center (ABRC, https://abrc.osu.edu/). The coding sequence (CDS) of *CKX5* was cloned using reverse transcription-PCR from total RNA obtained from Arabidopsis seedlings. The PCR products were subsequently sequenced and introduced into the binary vector pCAMBIA1300, under the control of the super promoter, which consists of Cauliflower Mosaic Virus (CaMV) 35S promoter and the mannopine synthase gene promoter. This resulting construct was designated as 1300-CKX5. To create transgenic lines of CKX5OE, 1300-CKX5 was transformed into *Arabidopsis* Col-0 genome using the *Agrobacterium*-mediated flower-dip method [16]. The plants were grown in growth chamber under conditions of 16 h’ white light (∼100 μmol m^−2^ s^−1^) at 23 °C/8 h’ darkness at 20 °C, with 60% humidity. Seeds of *Arabidopsis* plants used for *B*. *cinerea* infection experiments were surface-sterilized and grown on plates with solid Murashige and Skoog (MS) medium for two weeks after vernalization. Then, the plates were transplanted to sterile soil and grown under a 12 h light/12 h dark photoperiod.

### Pathogen Bioassays

For pathogen infection, the manipulations about *B*. *cinerea* (strain B05.10) cultivation, preparation and inoculation of spore suspension, the analysis of disease symptoms were performed according to [13]. After culturation on potato dextrose agar (PDA) medium (Coolaber, Beijing) for 10 days, The *B*. *cinerea* spores were harvested and suspended in a half-strength potato dextrose broth (1/2 PDB) to achieve a final concentration of 2.5 × 10^5^ spores mL^-1^. Subsequently, 4 μL droplet of the spore suspension was placed onto the adaxial side of the leaves of intact plants that were 4 weeks old. After the inoculations, all the plants were placed under sealed transparent hoods in a high humidity environment.

Two days after the *B*. *cinerea* infection, the symptoms on the infected leaves were examined. Lesion diameters on the leaves were measured using Image J software. Following the method described by [17], the disease symptoms on the inoculated leaves were recorded and classified into four groups: class I for lesion diameters less than 2 mm, class II for 2-mm lesions with chlorosis, class III for 2–4 mm lesions with chlorosis, and class IV for lesions with a spread greater than 4 mm.

### Quantitative real-time reverse transcription PCR (qRT-PCR)

After inoculation of *B*. *cinerea* spores and mock solution, the leaves at corresponding time points were collected and frozen in liquid nitrogen for total RNA extraction and qRT-PCR. This whole process proceeded according to [13]. Primers used for reference genes and genes of interest are listed in S1 Table.

### Yeast one-hybrid screening

The Y1H screening assays were performed using a Matchmaker Gold Yeast One-Hybrid Library Screening System. The prey pool containing TFs cDNA library inserted into the prey vector pGADT7 was obtained from Dr. Huayan Zhao (Hubei University). Five *CKX5* promoter fragments were separately inserted into pAbAi to construct the pAbAi-baits. Then the pAbAi-bait plasmids were linearized and transformed into Y1HGold. The colonies were selected on the synthetically defined medium lacking uracil (SD-Ura). After determining the minimal inhibitory concentrations of aureobasidin A (AbA) for the bait strains, the linear prey vector pool was co-transformed into the bait yeast strains and selected on synthetic dextrose (SD)/-Leu/AbA plates. The plasmids in the positive colonies were extracted and sequenced to confirm the corresponding gene number in TAIR (The Arabidopsis Information Resource, https://www.arabidopsis.org/). The primers used for cloning *CKX5* promoter fragments are listed in S2 Table.

### Pair-wise yeast one-hybrid

The coding DNA of the selected TFs were cloned from *Arabidopsis* genome by PCR and inserted into the MCS of pGADT7 to make pGADT7-TFs. Then linearized pGADT7-TFs were separately transformed into the corresponding bait-strains and grown on SD-Leu/AbA medium. The primers used to amplify TFs are listed in S3 Table.

## Results

### Regulation of *CKX* family genes by *B*. *cinerea* infection

**Fig 1:**
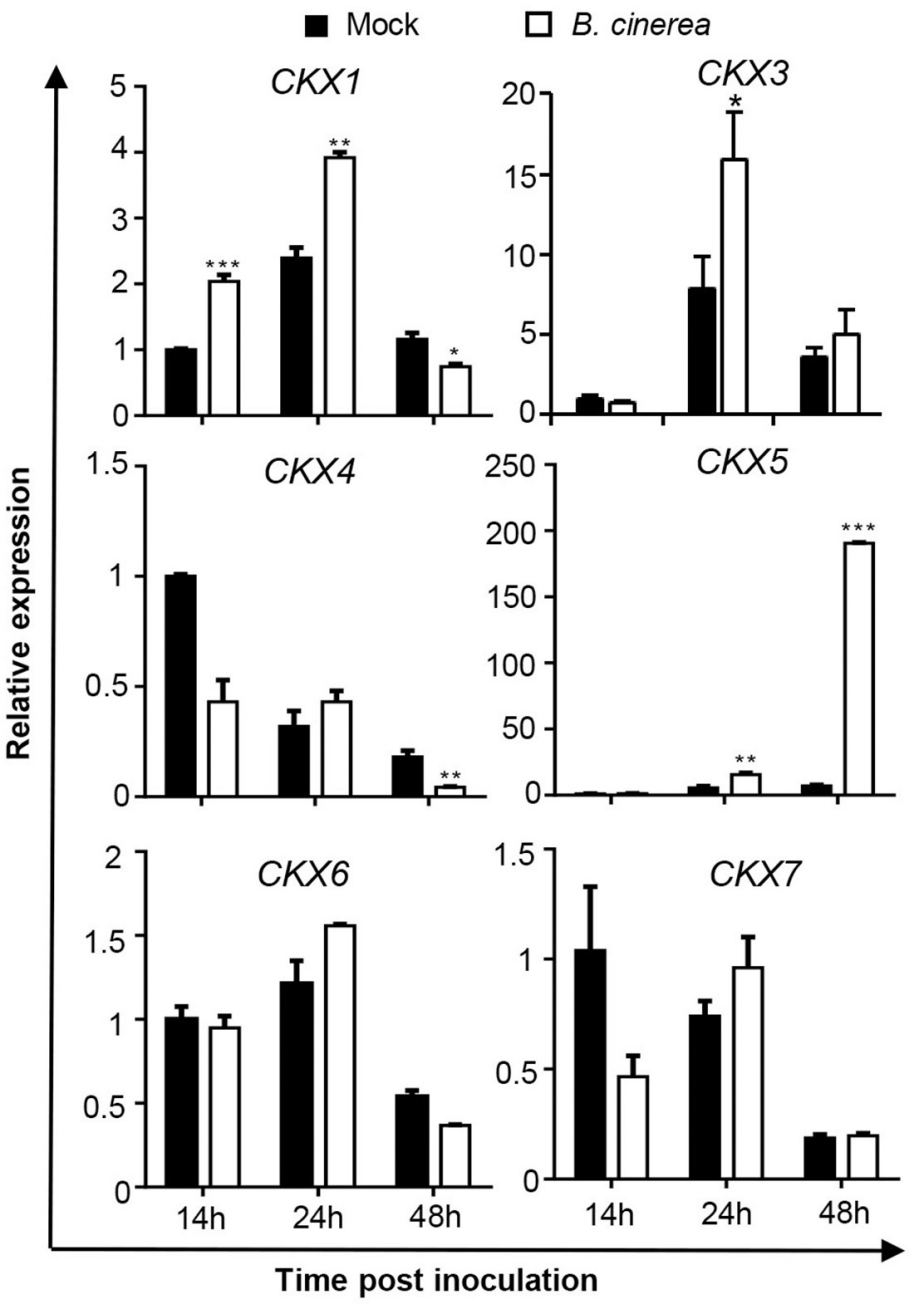
Expression profiles of cytokinin oxidase (CKX) genes in *Arabidopsis* plants after *Botrytis cinerea* inoculation. Transcript levels of *CKX* family were determined by qRT-PCR in 4-weeks-old leaves inoculated with *B*. *cinerea* spores at different time-points, and taking leaves inoculated with the same amount of 1/2 PDB solution as the mock treatment. Expression levels in the mock leaves at 14 h post inoculation were set to a value of 1. All data were normalized to the expression of *EXP* (At4g26410). Asterisks indicate significant differences between *B*. *cinerea* and mock-treated samples at the same time-point (two-tailed Student’s *t*-test: *P < 0.05, **P < 0.01, ***P < 0.001). Error bars are standard deviations (n = 3). Three independent experiments were performed with a similar outcome; results from one representative experiment are shown.

In our previous work, it was shown that expression of *CKX5* was markedly induced in *B*. *cinerea*-infected leaves and distant leaves of the same plant [13]. In the present study, we analyzed transcript levels of the *Arabidopsis CKX* family by qRT‒PCR. *CKX2* is not shown here due to the undetectable expression in *B*. *cinerea*-infected and mock leaves. Based on the results, *CKX* genes were differentially regulated by *B*. *cinerea* infection vs. the mock-treated leaves (Fig 1). Among them, *CKX1* responded rapidly, being upregulated at 14 h and 24 h post inoculation (hpi) but downregulated at 48 hpi. *CKX3* was only induced at 24 hpi. Consistent with our previous work [13], *CKX4* expression was reduced at 48 hpi. Markedly and consistently, the transcript level of *CKX5* was significantly increased after *B*. *cinerea* infection. The other two *CKX* genes, *CKX6* and *CKX7*, were not affected after *B*. *cinerea* inoculation. Combined with the *CKX5*:*GUS* analysis in [13], our qRT‒PCR results suggest an important role for *CKX5* in plant immunity to *B*. *cinerea* infection.

### *CKX5*-overexpressing *Arabidopsis* plants show resistance to *B*. *cinerea* infection

**Fig 2:**
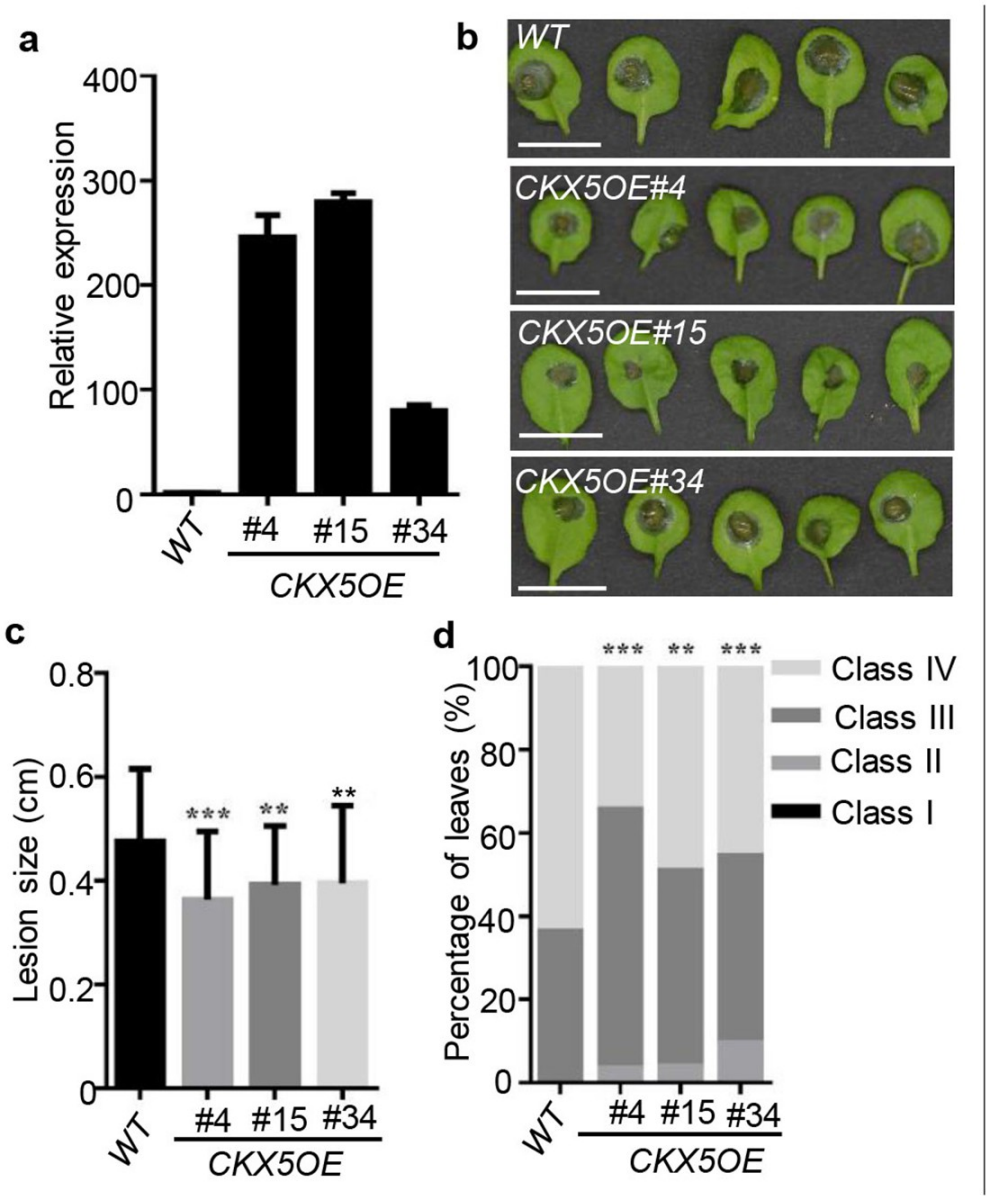
Transgenic *Arabidopsis* plants with overexpression of *CKX5* were more resistant to *Botrytis cinerea* infection. (**a)** Relative expression levels of *CKX5* in transgenic *Arabidopsis*. *WT*, wild type. *CKX5OE*, *CKX5*-overexpressing transgenic lines. (**b)** Leaves from 4-weeks-old WT and the three transgenic lines were photographed 2 days after *B*. *cinerea* inoculation. Scale bars = 1 cm. (**c)** Disease lesion size in the indicated genotypes. The values shown are the means ± SD (n = 50 inoculated leaves). Significant differences between WT and transgenic lines were analyzed by two-tailed Student’s *t*-test. **P < 0.01; ***P < 0.001. (**d)** Disease symptoms of leaves from the indicated genotypes. Class I, lesion < 2 mm; Class II, 2 mm lesion plus chlorosis; Class III, 2–4 mm lesion plus chlorosis; Class IV, lesion > 4 mm plus chlorosis. The distribution was calculated from 50 leaves. The significance of differences was analyzed by χ^2^-test. **P < 0.01; ***P < 0.001.

To further confirm the importance of *CKX5* in the plant-*B*. *cinerea* interaction, we generated transgenic *Arabidopsis* plants with much higher transcript levels of *CKX5* than wild-type (WT) plants (Figs 2a and S1). Then, four-week-old *CKX5*-overexpression lines were inoculated with *B*. *cinerea* spores, and disease symptoms were recorded 2 days later. The results shown in Fig 2b–2d clearly reveal much smaller lesions and less severe disease symptoms in leaves from the plants overexpressing *CKX5* compared with WT plants, indicating that the *CKX5*-overexpressing plants were more resistant to *B*. *cinerea*.

### Screening of transcription factors binding to the *CKX5* promoter by yeast one-hybrid assays

**Fig 3:**
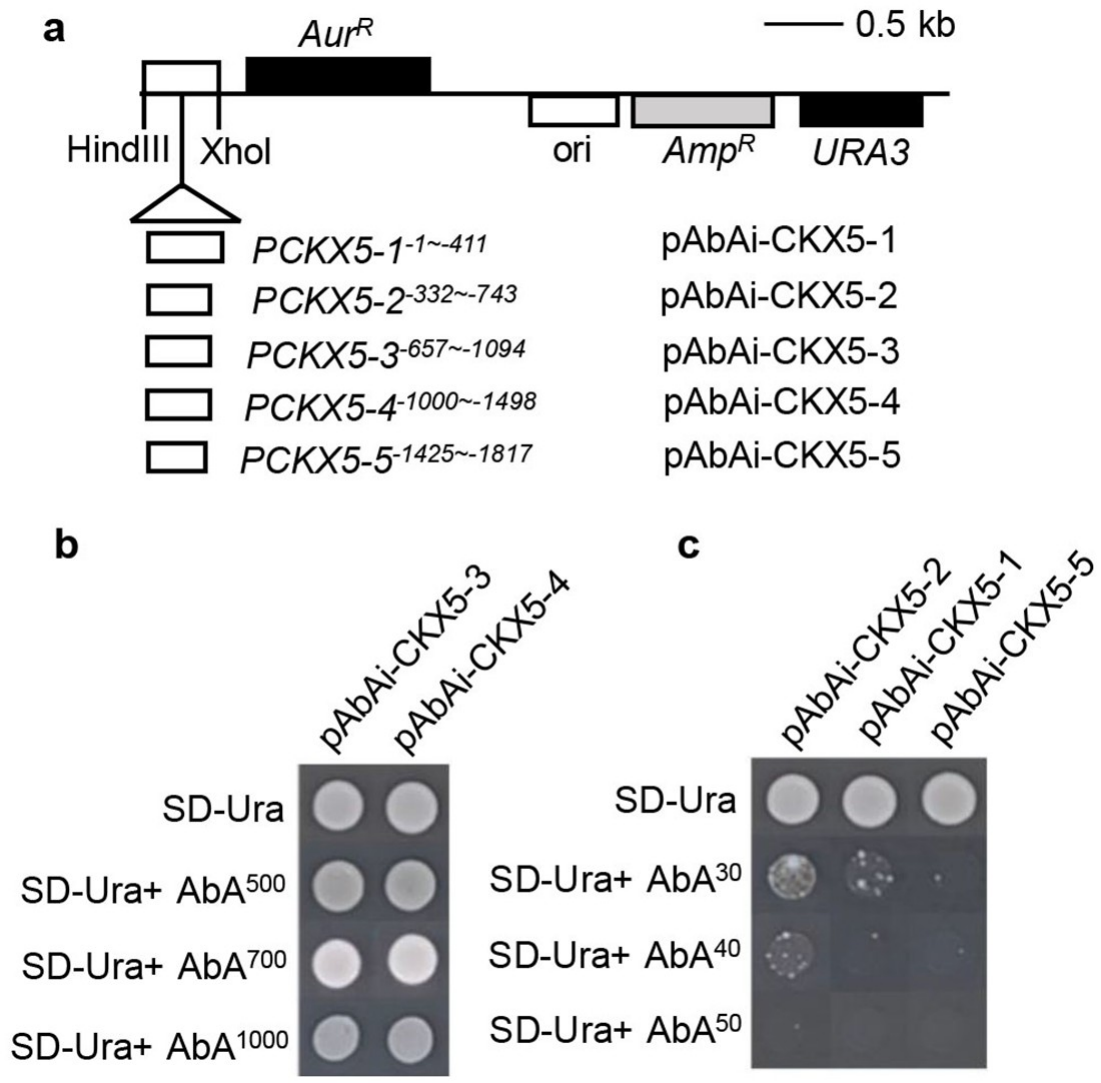
Generation of bait strains for yeast one-hybrid (Y1H) screening. **(a)** Physical map of the bait plasmids used for integration into yeast genome. Five short overlapping promoter fragments of *CKX5* were separately inserted into the vector of pAbAi through HindIII and XhoI restriction sites. *Aur*^*R*^, encodes aureobasidin A-resistant mutant of inositol phosphorylceramide synthase, confers fungal resistance to Aureobasidin A (AbA); *Amp*^*R*^, confers resistance to ampicillin, carbenicillin, and related antibiotics; *URA3*, encodes orotidine5’-phosphatedecarboxylase. (**b)** Determination the minimal inhibitory concentrations of AbA for the bait strains. The numbers on the right side of AbA represent the concentration of AbA with unit of ng/mL. SD-Ura means synthetically defined medium lacking uracil.

To determine which factor induces strong upregulation of *CKX5*, a yeast one-hybrid (Y1H) approach was performed to screen for TFs binding to the *CKX5* promoter sequence. Since yeast promoters are rather compact [18], the 1817 base pair (bp) sequence of the *CKX5* promoter was divided into five short overlapping segments of ~400 bp in length (Fig 3a). Each fragment was cloned into the pAbAi vector as a single copy to generate a bait construct, and then bait strains were created by integrating the pBait-AbAi plasmids into the Y1HGold yeast genome. Five kinds of bait strains were obtained, including pAbAi-CKX5-1 (from -1 to -411 bp of the promoter, P*CKX5-1*^−1~-411^), pAbAi-CKX5-2 (-332 to -743 bp, P*CKX5-2*^−332~-743^), pAbAi-CKX5-3 (-657 to -1094 bp, P*CKX5-3*^−657~-1094^), pAbAi-CKX5-4 (-1000 to -1498 bp, P*CKX5-4*^−1000~-1498^) and pAbAi-CKX5-5 (-1425 to -1817 bp, P*CKX5-5*^−1425~-1817^). To determine whether the bait strains show background growth on the synthetically defined medium lacking uracil (SD-Ura), a titration was performed using aureobasidin A (AbA). As illustrated in Fig 3b, endogenous activation of pAbAi-CKX5-3 and pAbAi-CKX5-4 was so strong that the addition of up to 1000 ng/mL AbA was still not sufficient to inhibit background growth. Fig 3c indicates that the optimal concentrations of AbA used for selection of pAbAi-CKX5-2, pAbAi-CKX5-1 and pAbAi-CKX5-3 were 50 ng/mL, 40 ng/mL and 30 ng/mL, respectively; therefore, these three bait strains were used for ensuing one-hybrid screens.

An *Arabidopsis* cDNA library was transformed into the bait strains, followed by selection on SD medium lacking leucine (SD-Leu) with corresponding concentrations of AbA. Prey TFs from the positive colonies were chosen after checking that their expression was regulated by *B*. *cinerea* infection using the microarray platform GENEVESTIGATOR (https://genevestigator.com/gv/). Together, 13 potential TFs responding to *B*. *cinerea* infection according to GENEVESTIGATOR were selected for further analysis (S4 Table).

**Fig 4:**
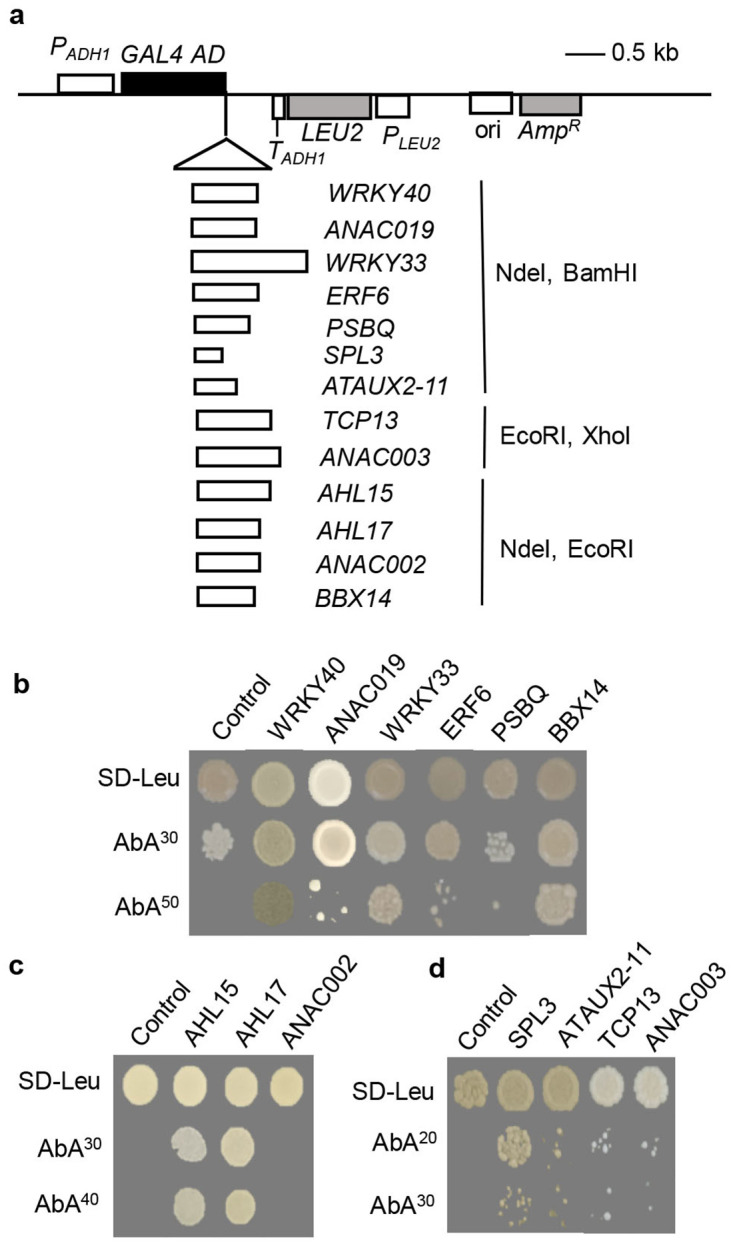
Confirmation of the 13 screened transcription factors (TF) with pair-wise yeast one hybrid. **(a)** Coding sequences (CDS) of the screened 13 TFs were separately cloned into the prey vector pGADT7 AD in frame with the activation domain (AD). The corresponding gene name and restriction sites used for cloning were shown. *P*_*ADH1*_, constitutive ADH1 promoter; *GAL4 AD*, encodes GAL4 activation domain; *LEU2*, nutritional marker for selection in yeast. (**b-d)** The prey vectors were transformed into the corresponding bait strains and grown on SD medium lacking leucine (SD-Leu) with corresponding concentrations of AbA. The control stains contained empty pGADT7 AD and bait vector.

Next, pair-wise yeast one-hybrid assays were performed after the coding sequences of the 13 TFs were separately inserted into the pGADT7 vector (Fig 4a). The results revealed that ANAC019 (NAC DOMAIN-CONTAINING PROTEIN 19, AT1G52890), WRKY40 (WRKY DNA-BINDING PROTEIN 40, AT1G80840), WRKY33 (WRKY DNA-BINDING PROTEIN 33, AT2G38470), ERF6 (ETHYLENE RESPONSE FACTOR, AT4G17490), BBX14 (B-BOX DOMAIN PROTEIN 14, AT1G68520) and PSBQ (PHOTOSYSTEM II SUBUNIT Q, AT4G05180) could bind P*CKX5-2*^−332~-743^ strongly (Fig 4b). AHL15 (AT-HOOK MOTIF NUCLEAR-LOCALIZED PROTEIN 15, AT3G55560) and AHL17 (AT5G49700) strongly bound to P*CKX5-1*^−1~-411^ (Fig 4c). Binding of SPL3 (SQUAMOSA PROMOTER BINDING PROTEIN-LIKE 3, AT2G33810) to P*CKX5-5*^−1425~-1817^ was strong, whereas that of ATAUX2-11 (AT5G43700), TCP13 (PLASTID TRANSCRIPTION FACTOR 1, AT3G02150) and ANAC003 (NAC DOMAIN CONTAINING PROTEIN 3, AT1G02220) was weak (Fig 4d).

**Fig 5:**
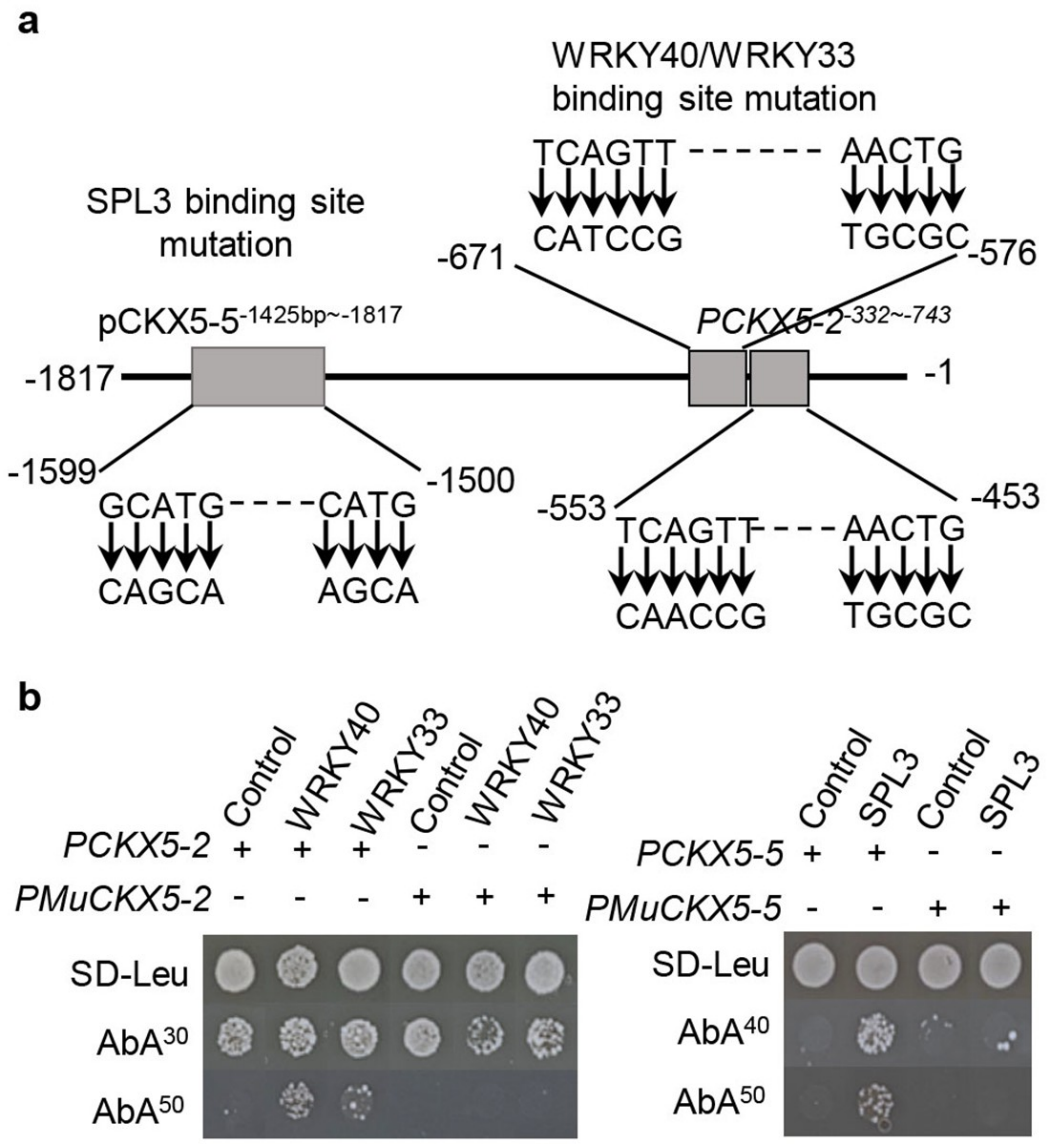
Mutation of the binding sequences repressed the interactions between WRKY40, WRKY33, SPL3 and *CKX5* promoter fragments. (**a)** Physical map of the *CKX5* promoter illustrating the changes of binding sites for WRKY40, WRKY33 and SPL3. The arrows showed how the original sequences were changed. (**b)** Determination of the interactions between the three transcription regulators and the mutated promoter of CKX5 with yeast one-hybrid assays. Y1H Gold strains successfully transformed with corresponding vectors were grown on the SD-Leu/AbA plates at 30 °C for 3–5 days. *PCKX5-2* or *PCKX5-5* represents the original fragment of *CKX5* promoter. *PMuCKX5-2* or *PMuCKX5-5* means the fragments with mutated binding sites. The numbers on the right side of AbA represent the concentration of AbA with unit of ng/mL.

To further confirm the binding of these TFs to the *CKX5* promoter sequence, JASPAR (https://jaspar.genereg.net/) was used to predict binding sites for the TFs, but only binding sites for WRKY40, WRKY33 and SPL3 were found. Then, the predicted binding sequences of the three TFs were mutated as depicted in Fig 5a and used as bait sequences for yeast one-hybrid assays. The growth of yeast colonies carrying mutated bait sequences was completely arrested on SD-Leu medium with 50 ng/mL AbA; however, the growth of yeast colonies with original bait sequences was not when transformed with corresponding TFs (Fig 5b). This further suggested that WRKY40, WRKY33 and SPL3 interact with the *CKX5* promoter by binding at corresponding sites.

### The screened TFs are involved in plant immunity to *B*. *cinerea*

**Fig 6:**
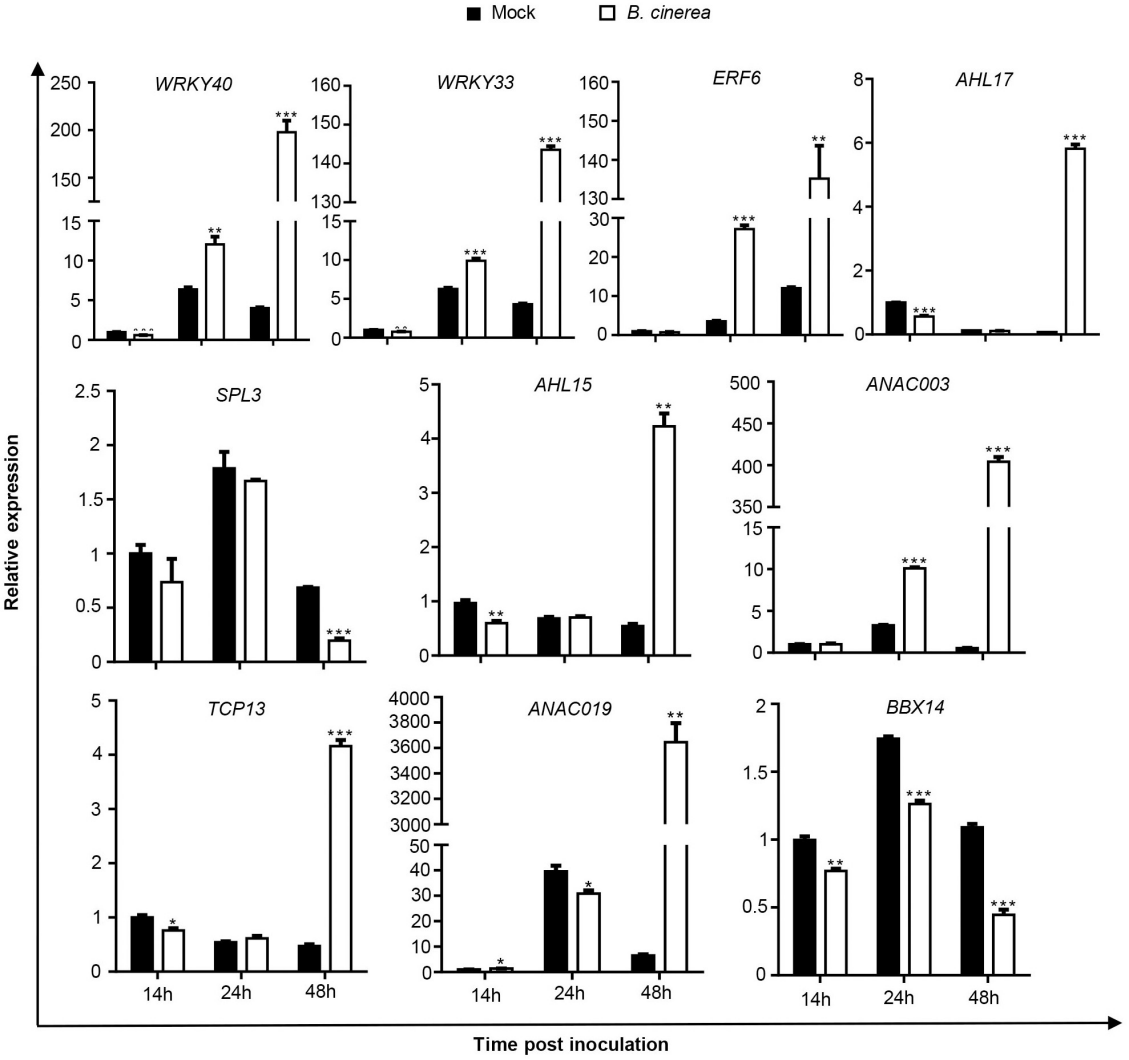
Expression levels of selected transcription regulator genes in response to *Botrytis cinerea*. Transcript accumulations of the genes were determined by real-time qPCR in 4-weeks-old wild type *Arabidopsis* leaves inoculated with *B*. *cinerea* at different time-points. Leaves inoculated with same amount of 1/2 PDB solution as mock. Expression levels in the mock leaves at 14 h post-inoculation (hpi) were set to a value of 1. Error bars are standard deviations (n = 3). Differences are significant at P < 0.05 (*), P < 0.01 (**) and P < 0.001 (***) by Student’s *t*-test.

To investigate whether the TFs screened by yeast one-hybrid are truly involved in the response to *B*. *cinerea* infection, transcript levels of *WRKY40*, *ARKY33*, *ERF6*, *AHL17*, *SPL3*, *AHL15*, *ANAC003*, *TCP13*, *ANAC019* and *BBX14* were analyzed by qRT‒PCR in *Arabidopsis* leaves inoculated with *B*. *cinerea* at 14, 24 and 48 hpi (Fig 6). According to the data, the transcript levels of *WRKY40*, *WRKY33*, *ERF6* and *ANAC003* were strongly upregulated after *B*. *cinerea* inoculation, at levels 40-fold, 29-fold, 13.4-fold and 400-fold of mock treatments at 48 hpi. Conversely, *SPL13* and *BBX14* expression was repressed. For *AHL17*, *AHL15*, *TCP13* and *ANAC019*, expression levels were downregulated at 14 hpi or 24 hpi and then significantly upregulated at 48 hpi. The transcript level of *ANAC019* in *B*. *cinerea*-infected leaves was 450 times that in mock leaves at 48 hpi.

**Fig 7:**
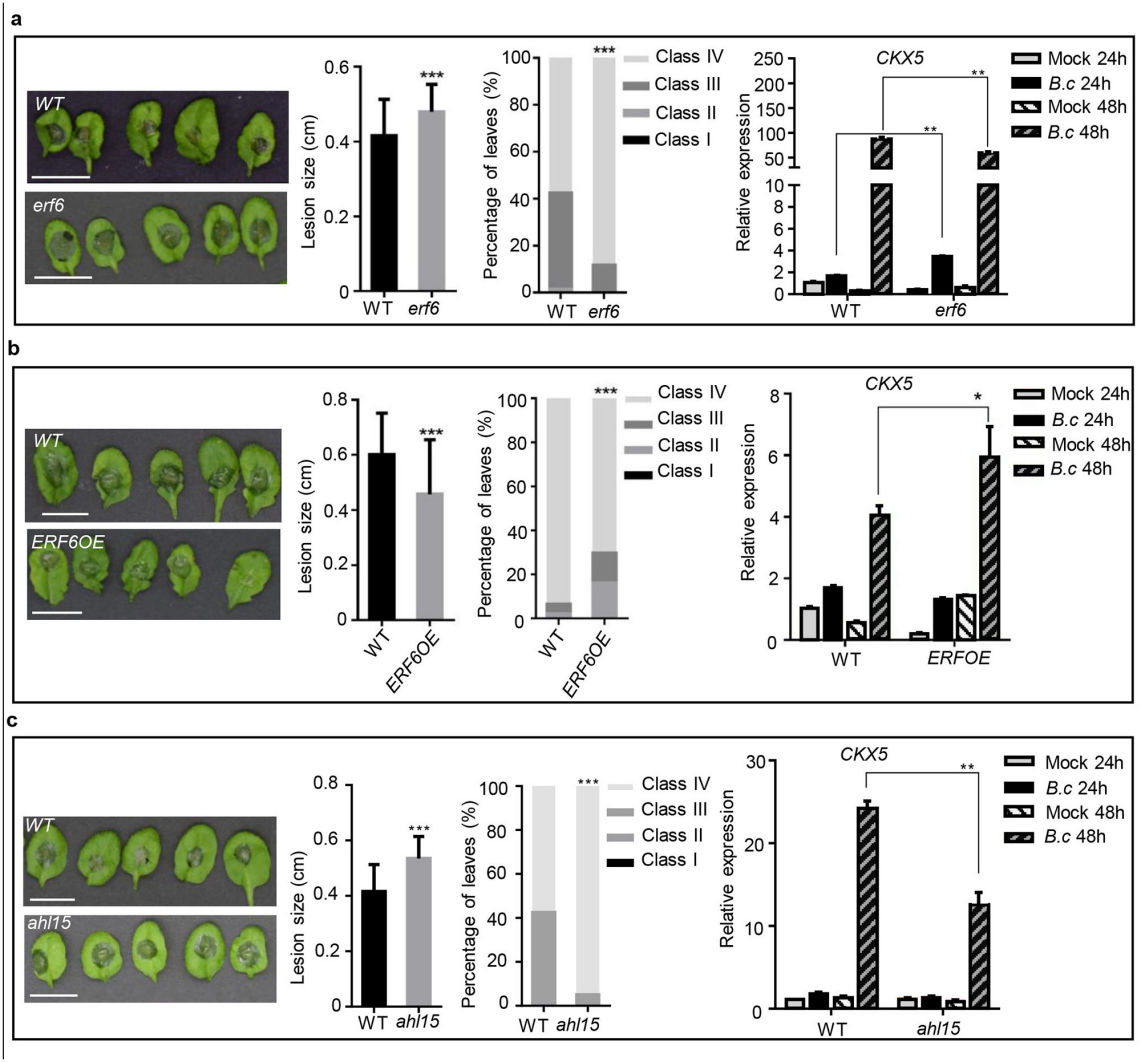
Alteration of in *vivo EFR6* or *AHL15* levels changed the susceptibility of *Arabidopsis* plants and the response of *CKX5* to *Botrytis cinerea* infection. **(a-c)** Leaves of 4-weeks-old wild type (WT) and *erf6* mutant, *ERF6* overexpression plants (*ERF6OE*), *ahl15* mutant were inoculated with *B*. *cinerea* spores and the disease symptoms were analyzed two days later. Scale bars in photographs of the infected leaves are 1 cm. The values shown for disease lesion size in the indicated genotypes are means ± SD (n = 50 inoculated leaves). Asterisks indicate significant differences between WT and mutant or transgenic plants (two-tailed Student’s *t*-test, **P < 0.01; ***P < 0.001). The distribution of leaves in the class I-IV was calculated from 50 leaves. Class I, lesion < 2 mm; Class II, 2 mm lesion plus chlorosis; Class III, lesion 2–4 mm plus chlorosis; Class IV, lesion > 4 mm plus chlorosis. The significance of differences was analyzed by χ^2^-test. **P < 0.01; ***P < 0.001. Meanwhile, *CKX5* expression levels were analyzed in leaves of the four genotypes at 24 h and 48 h post-inoculation with *B*. *cinerea* or mock solution. Expression values in WT leaves with mock treatment were set to a value of 1. Bars are the means ± SD (n = 3). Three independent experiments were performed with similar outcomes; results from one representative experiment are shown. Asterisks indicate significant differences between WT and *erf6*, *ERF6OE*, or *ahl15* with same treatments (two-tailed Student’s *t*-test, *P < 0.05; **P < 0.01).

Next, we employed mutant *erf6*, *ERF6*-overexpressing (*ERF6OE*) and mutant *ahl15* plants to verify involvement of the TFs in plant immunity to *B*. *cinerea* and regulation of *CKX5*. As shown in S2 Fig, mutant *erf6* carries a T-DNA insertion in the exon of *ERF6* and displayed strongly reduced expression of *ERF6*. Overexpression of *ERF6* was induced by β-estradiol, and *AHL15* expression could not be detected in *ahl15*, which has a T-DNA insertion in the second exon of *AHL15*. Leaves of these plants were inoculated with *B*. *cinerea* spores; disease symptoms were recorded 48 h later, and expression levels of *CKX5* in leaves infected with *B*. *cinerea* in WT, *erf6*, *ERF6OE* and *ahl15* at 24 and 48 hpi were assessed via qRT‒PCR (Fig 7). The results provided in Fig 7a clearly illustrate that *ERF6*-knockdown mutants developed more severe disease symptoms than WT plants, suggesting the enhanced susceptibility of *erf6* to *B*. *cinerea*. In addition, *ERF6*-overexpressing plants had less severe symptoms than WT plants (Fig 7b). Altogether, the results indicate the positive role of *ERF6* in plant immunity to *B*. *cinerea* infection. Analysis of *CKX5* transcript levels in *B*. *cinerea*-infected leaves revealed more pronounced transcript accumulation of *CKX5* at 24 hpi but lower transcript levels at 48 hpi in *erf6* mutant leaves than in wild-type leaves (Fig 7a). In contrast, the abundance of the *CKX5* transcript was slightly higher in *ERF6OE* than in WT in the presence of *B*. *cinerea* infection at 48 hpi (Fig 7b). These results suggest regulation of *CKX5* by ERF6during *B*. *cinerea* infection. The effect of mutation of *AHL15* is shown in Fig 7c. The *ahl15* mutant exhibited more severe disease symptoms than WT, indicating that enhanced susceptibility of *Arabidopsis* to *B*. *cinerea* may be induced by a lack of *AHL15*. Furthermore, knockout of *AHL15* resulted in significant repression of *CKX5* transcript in *ahl15* leaves after challenging them with *B*. *cinerea* when compared to WT, suggesting the possible transcript regulation of *CKX5* by AHL15 in *B*. *cinerea*-infected leaves.

## Discussion and conclusions

With qRT‒PCR and analysis of the reporter line *CKX5*:*GUS*, we previously showed that *Arabidopsis CKX5* expression is remarkably induced in leaves infected with *B*. *cinerea*, especially when the leaves show some necrotic areas due to *B*. *cinerea* [13]. Such high expression of *CKX5* was also detected in the distant leaves of the same plant not inoculated when *B*. *cinerea* successfully caused decay of part of the inoculated leaves. Therefore, we addressed the involvement of CKX5 in *Arabidopsis*-*B*. *cinerea* interaction and potential of TFs regulating *CKX5* in response to *B*. *cinerea* infection in the present study.

In *Arabidopsis thaliana*, there are seven distinct *CKX* genes (*CKX1*-*CKX7*) that encode cytokinin oxidase/dehydrogenases responsible for the breakdown of CKs *in vivo*, though their preferred substrates and enzyme activity differ under different conditions [19,20]. In our study, *CKX* genes were found to respond differently to *B*. *cinerea* infection (Fig 1). The most prominently changed *CKX* gene was *CKX5*, especially at 48 hpi, when most of the inoculated leaves had decayed. Transgenic *Arabidopsis* plants overexpressing *CKX5* showed increased resistance to *B*. *cinerea* compared with WT plants (Fig 2), which does not agree with reported results that tomato leaves overexpressing *CKX4* are more sensitive to *B*. *cinerea* infection [12]. This may be explained as follows. First, *CKX4* and *CKX5* responded differently to *B*. *cinerea* infection (Fig 1; Ref. 13). More importantly, CK has a dual role in *B*. *cinerea* biology [14,15]: (1) CK strongly inhibits *B*. *cinerea* growth and virulence by targeting the cytoskeleton and cellular trafficking in high nutritional availability; (2) CK increases the metabolism of *B*. *cinerea* under energy-restrictive conditions. Additionally, during the battle between plant and pathogen, necrotrophic fungal pathogens have been reported to cause the formation of green areas termed green necronissia in leaves, which might be induced by CKs [21]. Thus, our current results regarding CKX5 suggest that to block the benefits that *B*. *cinerea* receive from CKs, CKX5 is deployed to manipulate CK levels when *B*. *cinerea* successfully attacks the host.

By yeast one-hybrid screens, some TFs were found to bind to the promoter of *CKX5*. qPCR analysis showed that the transcript levels of selected TFs were significantly changed during *B*. *cinerea* infection (Fig 6). Among them, *WRKY40*, *WRKY33*, *ERF6* and *ANAC003* responded in a manner similar to *CKX5* in leaves inoculated with *B*. *cinerea*. WRKY proteins are well-known TFs that function in plant immunity to biotic stress. WRKY40 interacts with WRKY18 and WRKY60 to function in a pattern of overlapping, antagonistic, and distinct roles in plant responses to *Pseudomonas syringae* and *B*. *cinerea* [22]. WRKY33 is a key transcriptional regulator in plant immunity, and it has been indicated that *B*. *cinerea* B05. 10 promotes disease by suppressing WRKY33-mediated host defenses [23,24]. ERF6 is a positive regulator of JA/ET-mediated defense against *B*. *cinerea* in *Arabidopsis* [25]. ANAC003 is a senescence-related protein that activates expression of senescence-associated genes (SAGs), resulting in plant senescence [26]. The genes encoding another four TFs, including AHL15, AHL17, TCP13 and ANAC019, were strongly induced at the late stage of *B*. *cinerea* infection (48 hpi). Transient expression of *AHL15* in protoplasts blocks pathogen-associated molecular pattern (PAMP)-associated gene expression, suggesting its important role in plant immunity [27]. ANAC019 has been indicated to function as a transcription activator to regulate JA-induced expression of defense genes [28]. Most of these TFs are involved in plant defense against pathogens. *ERF6*-overexpressing plants and *ERF6* and *AHL15* mutants further proved the upregulation role of ERF6 and AHL15 in *CKX5* during *B*. *cinerea* infection (Fig 7). However, the results indicate that strong upregulation of *CKX5* was not only controlled by ERF6 and AHL15. It is likely that most of the TFs mentioned above are involved in this process. We therefore propose that *CKX5* is manipulated by TFs belonging to the plant defense system to counteract the necrotrophic phase of infection by *B*. *cinerea*.

## Supporting information

S1 Fig Phenotypes of wild type (*WT*) and transgenic *Arabidopsis* plants overexpressing *CKX5* (*CKX5OE*). (a) Seed germination assays on medium containing 30 mg/L hygromycin confirm the homozygosity of the transgenic lines. (b) 4 weeks-old wild type and transgenic plants *CKX5OE* grown under photoautotrophic conditions in soil. Bars in (a) and (b) are 1 cm.
(JPG)

S2 Fig T-DNA insertion mutants for *ERF6*, *AHL15* and *ERF6*-overexpressing (*ERF6OE*) plants. (a) Diagram for T-DNA insertion mutants of *ERF6* and *AHL15*. (b) qRT-PCR analysis of *ERF6* transcript levels in *erf6* and *ERF6OE*, *AHL15* transcript level in *ahl15*. *ERF6* overexpression is β-estradiol induced. 4 weeks old wild type (WT) and *ERF6OE* plants were sprayed with 100 μM β-estradiol for 2 days, three times per day. Then the leaves were used for RNA extraction. Expression levels in WT leaves before spaying (0 h) were set to a value of 1. All data were normalized to the expression of *EXP* (At4g26410). Error bars are standard deviations (n = 3). n. d., not detected. Three independent experiments were performed with a similar outcome; results from one representative experiment are shown.
(JPG)

S1 Table Primers used for quantitative real-time PCR. (DOCX)

S2 Table Primers used for cloning *CKX5* promoter fragments. (DOCX)

S3 Table Primers used to amplify the coding sequences of selected transcription factors. (DOCX)

S4 Table The information for selected transcription factors screened by yeast one-hybrid. (DOCX)
