# Supplementary figures and images for: Cytokinin oxidase gene CKX5 is modulated in the immunity of Arabidopsis to Botrytis cinerea
Source: PLoS One. 2024 Mar 13;19(3):e0298260. doi: 10.1371/journal.pone.0298260 (PMC10936862; doi:10.1371/journal.pone.0298260)

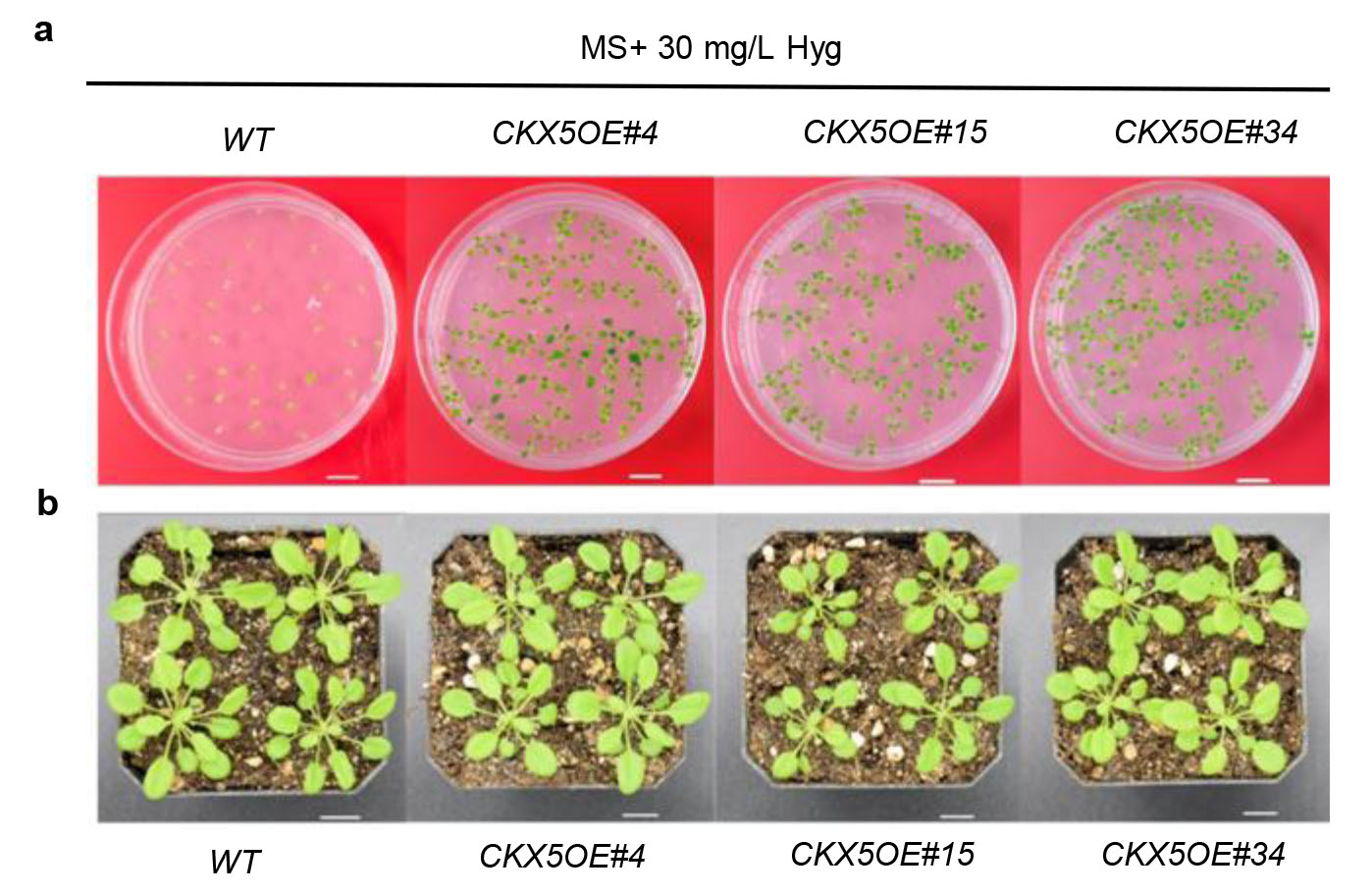

Supplement: S1 Fig — (a) Seed germination assays on medium containing 30 mg/L hygromycin confirm the homozygosity of the transgenic lines. (b) 4 weeks-old wild type and transgenic plants CKX5OE grown under photoautotrophic conditions in soil. Bars in (a) and (b) are 1 cm. (JPG) [file pone.0298260.s001.jpg]

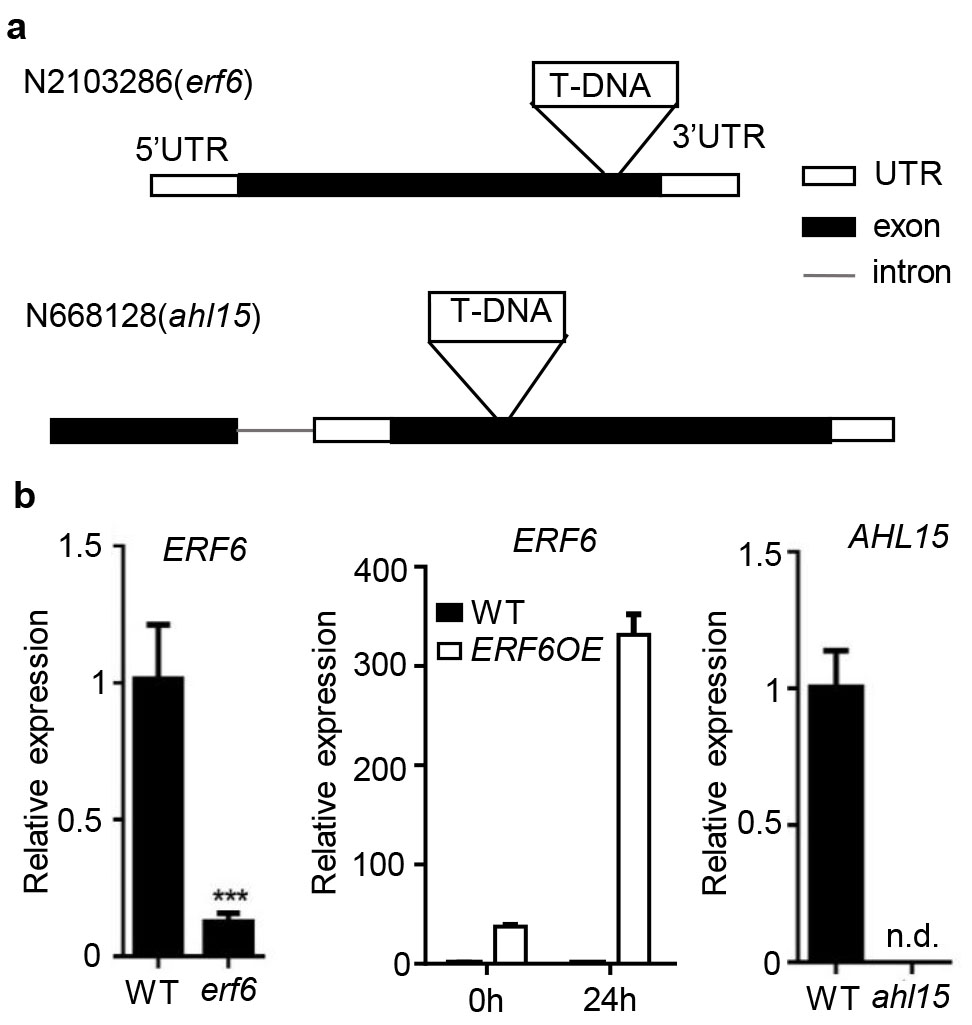

Supplement: S2 Fig — (a) Diagram for T-DNA insertion mutants of ERF6 and AHL15. (b) qRT-PCR analysis of ERF6 transcript levels in erf6 and ERF6OE, AHL15 transcript level in ahl15. ERF6 overexpression is β-estradiol induced. 4 weeks old wild type (WT) and ERF6OE plants were sprayed with 100 μM β-estradiol for 2 days, three times per day. Then the leaves were used for RNA extraction. Expression levels in WT leaves before spaying (0 h) were set to a value of 1. All data were normalized to the expression of EXP (At4g26410). Error bars are standard deviations (n = 3). n. d., not detected. Three independent experiments were performed with a similar outcome; results from one representative experiment are shown. (JPG) [file pone.0298260.s002.jpg]
