# Supplementary material for: Cytokinin oxidase gene CKX5 is modulated in the immunity of Arabidopsis to Botrytis cinerea
Source: PLoS One. 2024 Mar 13;19(3):e0298260. doi: 10.1371/journal.pone.0298260 (PMC10936862; doi:10.1371/journal.pone.0298260)
Supplement: S1 Table — (DOCX) [file pone.0298260.s003.docx]

**S1 Table.** Primers used for quantitative real-time PCR

| **Gene** | **Accession Number** | **Forward primer (5’-3’)** | **Reverse primer (5’-3’)** |
| --- | --- | --- | --- |
| EXP | AT4G26410 | GAGCTGAAGTGGCTTCCATGAC | GGTCCGACATACCCATGATCC |
| CKX1 | AT2G41510 | ACAGAGGAAACAAGCCTACGACCCT | GCCTTTGACTTTGCGAGTTGGATGG |
| CKX3 | AT5G56970 | AGCGGTCCTGTTCTTGTTTATCCT | ACCTCGGACCAAAATGTCTAACCC |
| CKX4 | AT4G29740 | ATAAAGGCTCAACCAGCCCC | ACGTCATGTTCACGACGACA |
| CKX5 | AT1G75450 | ACGGACGGTGAAGAGACACAGA | CCTGTGTTGCGTGGTGAGGA |
| CKX6 | AT3G63440 | GCATCTCCAGGGTCGGCAGG | ACTGCTTCAACCCTATCCCTGCTTCT |
| CKX7 | AT5G21482 | CACCAGAGCTAGGGTTTTGC | CATCGAACTCGGTGTATACTACTCTT |
| WRKY40 | AT1G80840 | GGGATCAAATCAGCCCTCCC | TCCGTTGAGCTACTCTCCGA |
| WRKY33 | AT2G38470 | GTGGGAGTGAACCTGAAGCA | TGCACTACGATTCTCGGCTC |
| ERF6 | AT4G17490 | GGCGATTCTGAATTTCCCGC | CCCAGTCATCTATCGCCGTC |
| AHL17 | AT5G49700 | ACCGACCCTCCTATGAGTCC | TAGCTACAGAGCCAGAGCCA |
| SPL3 | AT2G33810 | TGCCAGTTTCATGCCAAAGC | GCCTTCTCTCGTTGTGTCCA |
| AHL15 | AT3G55560 | TGTCGGAGAGCCATCAACAC | TCGCAACATTCCCTACCCAC |
| ANAC003 | AT1G02220 | GAGACACGAGCCATGTCGAT | CTGCTGATCACCTCTGCCAT |
| TCP13 | AT3G02150 | CGACGAGCTACCTCCGTTAC | TTAAACCCGAGTTGGGTCGG |
| ANAC019 | AT1G52890 | GTTCCACGGTCTTGCGGATA | CCGTCTTCAGGTAGCCACAG |
| BBX14 | AT1G68520 | GCCAACAACGCCTCTACACT | CAAGTTGCCAAGTCTCAGTTGC |
