# Supplementary material for: Cytokinin oxidase gene CKX5 is modulated in the immunity of Arabidopsis to Botrytis cinerea
Source: PLoS One. 2024 Mar 13;19(3):e0298260. doi: 10.1371/journal.pone.0298260 (PMC10936862; doi:10.1371/journal.pone.0298260)
Supplement: S2 Table — (DOCX) [file pone.0298260.s004.docx]

**S2 Table.** Primers used for cloning *CKX5* promoter fragments

| **Fragment** | **Forward primer (5’-3’)** | **Reverse primer (5’-3’)** |
| --- | --- | --- |
| PCKX5-1^-1~-411^ | AAGCTTACACGGTTCTTTTTTGGTTT | CTCGAGGAAACAAGAATCAAGATTGAGGA |
| PCKX5-2^-332~-743^ | AAGCTTGGGGACCAATCAAAATGG | CTCGAGAAAGAGTTGTACCGGAGAAGA |
| PCKX5-3^-657~-1094^ | AAGCTTGAGTTTGTGTTATTACCGTGC | CTCGAGAACCTTGATTTGACTATGGAC |
| PCKX5-4^-1000~-1498^ | AAGCTTTATCGACCCACCAAACTAATC | CTCGAGCACATTTGGATACGGAGAA |
| PCKX5-5^-1425~-1817^ | AAGCTTATCGACAAAGAGCAAATTATGAA | CTCGAGATTTTGGATTTCTGAGTAATG |

Note: the sequences underlined are cleavage sites of HindIII and XhoI.
