# Supplementary material for: Cytokinin oxidase gene CKX5 is modulated in the immunity of Arabidopsis to Botrytis cinerea
Source: PLoS One. 2024 Mar 13;19(3):e0298260. doi: 10.1371/journal.pone.0298260 (PMC10936862; doi:10.1371/journal.pone.0298260)
Supplement: S3 Table — (DOCX) [file pone.0298260.s005.docx]

**S3 Table.** Primers used to amplify the coding sequences of selected transcription factors.

| **Gene** | **Accession Number** | **Forward primer (5’-3’)** | **Reverse primer (5’-3’)** |
| --- | --- | --- | --- |
| WRKY40 | AT1G80840 | CATATGGATCAGTACTCATCCTCTTTGG | GGATCCCTATTTCTCGGTATGATTCTGTTG |
| WRKY33 | AT2G38470 | CATATGGCTGCTTCTTTTCTTACAATGGACAATAGC | GGATCCTCAGGGCATAAACGAATCGAAAAATGAGGT |
| ERF6 | AT4G17490 | CATATGGCTACACCAAACGAAGTATCAGCTCTTTTC | GGATCCTCAAACAACGGTCAATTGTGGATAACCAAA |
| AHL17 | AT5G49700 | CATATGAAAGGTGAATACAGAGAGCAAAAGAGTAACGAAAT | GAATTCTTAGTATGGCGGTGGAGCTCTGGCTGTAGGGGCCC |
| SPL3 | AT2G33810 | CATATGAGTATGAGAAGAAGCAAAGCGGAAGGGAAG | GGATCCTTAGTCAGTTGTGCTTTTCCGCCTTCTCTC |
| AHL15 | AT3G55560 | CATATGGCGAATCCTTGGTGGGTAGGGAATGTTGCGATCGG | GAATTCTCAATACGAAGGAGGAGCACGAGGCGGAGGACCAC |
| ANAC003 | AT1G02220 | GAATTCGAAACTCCTGTGGGTTTAAGATTCTGTCCG | CTCGAGTCAAGTTCTTGAGATGGAAGAACATAGGTG |
| TCP13 | AT3G02150 | GAATTCAATATCGTCTCTTGGAAAGATGCAAACGAC | CTCGAGTCACATATGGTGATCACTTCCTCTACTTGT |
| ANAC019 | AT1G52890 | CATATGGGTATCCAAGAAACTGACCCGTT | GGATCCTCACATAAACCCAAACCCACCAAC |
| BBX14 | AT1G68520 | CATATGATGAAAAGTTTGGCTAGTGCGGTTGGAGGG | GAATTCTTAGTGAGCAACACCAATTGAAGATCTCTT |
| PSBQ | AT4G05180 | CATATGGCTCAAGCAGTGACTTCGATGGCTGGCTTA | GGATCCTTAACCGAGCTTGGCAAGAACATTGTTCAA |
| AtAUX2-11 | AT5G43700 | CATATGGAAAAAGTTGATGTTTATGATGAGCTTGTT | GGATCCTTAAAGACCACCACAACCTAAACCTTTAAC |
| ANAC002 | AT1G01720 | CATATGTCAGAATTATTACAGTTGCCTCCAGGTTTCCGATT | GAATTCCTAGTAAGGCTTCTGCATGTACATGAACATATCCT |

Note: the sequences underlined are cleavage sites of restriction endonucleases used for cloning.
