# Supplementary material for: Cytokinin oxidase gene CKX5 is modulated in the immunity of Arabidopsis to Botrytis cinerea
Source: PLoS One. 2024 Mar 13;19(3):e0298260. doi: 10.1371/journal.pone.0298260 (PMC10936862; doi:10.1371/journal.pone.0298260)
Supplement: S4 Table — (DOCX) [file pone.0298260.s006.docx]

**S4 Table.** The information for selected transcription factors screened by yeast one-hybrid.

| Gene ID | Promoter fragment used for screening | Gene name | Description |
| --- | --- | --- | --- |
| AT1G52890 | *PCKX5-2* | *ANAC019* | Encodes a NAC transcription factor whose expression is induced by drought, high salt, and abscisic acid |
| AT1G80840 | *PCKX5-2* | *WRKY40* | Pathogen-induced transcription factor. Coexpression with *WRKY18* or *WRKY60* made plants more susceptible to both *P. syringae* and *B. cinerea*. |
| AT2G38470 | *PCKX5-2* | *WRKY33* | Regulates the antagonistic relationship between defense pathways mediating responses to P. syringae and necrotrophic fungal pathogens. |
| AT4G17490 | *PCKX5-2* | *ERF6* | Ethylene response factor 6, involved in the response to reactive oxygen species and light stress. |
| AT1G68520 | *PCKX5-2* | *BBX14* | B-BOX DOMAIN PROTEIN 14; Putative transcriptional regulatory protein. |
| AT4G05180 | *PCKX5-2* | *PSBQ* | Encodes the PsbQ subunit of the oxygen evolving complex of photosystem II. |
| AT3G55560 | *PCKX5-1* | *AHL15* | AT-HOOK MOTIF NUCLEAR-LOCALIZED PROTEIN 15, involved in negative regulation of innate immune response. |
| AT5G49700 | *PCKX5-1* | *AHL17* | AT-HOOK MOTIF NUCLEAR LOCALIZED PROTEIN 17, acts within defense response to fungus and response to abscisic acid. |
| AT1G01720 | *PCKX5-1* | *ANAC002* | ARABIDOPSIS NAC DOMAIN CONTAINING PROTEIN 2. Transcript level increases in response to wounding and abscisic acid. |
| AT2G33810 | *PCKX5-5* | *SPL3* | SQUAMOSA PROMOTER BINDING PROTEIN-LIKE 3, may directly regulate AP1, and is involved in regulation of flowering and vegetative phase change. |
| AT5G43700 | *PCKX5-5* | *ATAUX2-11* | AUXIN INDUCIBLE 2-11, Auxin inducible protein similar to transcription factors. |
| AT3G02150 | *PCKX5-5* | *TCP13* | A chloroplast trans-acting factor of the psbD light-responsive promoter. |
| AT1G02220 | *PCKX5-5* | *ANAC003* | NAC DOMAIN CONTAINING PROTEIN 3, functions as a negative regulator of the TDIF-PXY module and fine-tunes TDIF signaling in vascular development. |
